# Thrombus-specific manganese-based “nanobialys” for MR molecular imaging of ruptured plaque

**DOI:** 10.1186/1532-429X-14-S1-P136

**Published:** 2012-02-01

**Authors:** Dipanjan Pan, Shelton D Caruthers, Angana SenPan, Anne H Schmieder, Todd A Williams, Michael J Scott, Patrick J Gaffney, Samuel A Wickline, Gregory Lanza

**Affiliations:** 1 Medicine, Washington University, Saint Louis, MO, USA; 2 Surgery, Saint Thomas', London, UK

## Summary

Mathematical modeling studies have suggested that nonspherical, disc-shaped nanoparticles may have optimal intravascular flow and homing characteristics. In this study, we report the development of a fibrin-specific high-relaxivity bialy-shaped polymeric nanoparticle using porphyrin-chelated manganese. We anticipate that this agent would be highly effective for molecular imaging of microthrombi in ruptured atherosclerotic plaques.

## Background

Detection of microthrombi within fissures of vulnerable atherosclerotic plaques requires a sensitive molecular imaging contrast agent. Moreover, recent reports based on mathematical modeling suggest that nonspherical, disc-shaped nanoparticles could have improved intravascular flow characteristics, which may improve ligand-directed targeting. In light of the concern surrounding the use of gadolinium in patients with severe renal disease, the goal of this research was to develop a nonspherical fibrin-targeted manganese-based molecular imaging agent.

## Methods

A new class of manganese (III)-labeled, a toroidal-shaped, vascularly-constrained nanoparticles, “nano-bialys (MnNBs)”, was designed, synthesized, physically characterized, and evaluated for MR properties. Single slice inversion recovery and multi-echo spin echo sequences were used to calculate the ionic (per metal) and particulate (per particle) relaxivities from 7 serial dilutions of nanobialys at 1.5T and 25°C. Fibrin clots supported on silk suture suspended in PBS were targeted with MnNB or control (non-paramagnetic) NB to the fibrin clots with avidin-biotin interactions and fibrin-specific antibodies (NIB5F3). Magnetic resonance images (3T) of the clots were acquired using T1-weighted gradient echo techniques.

## Results

**Figure 1:**
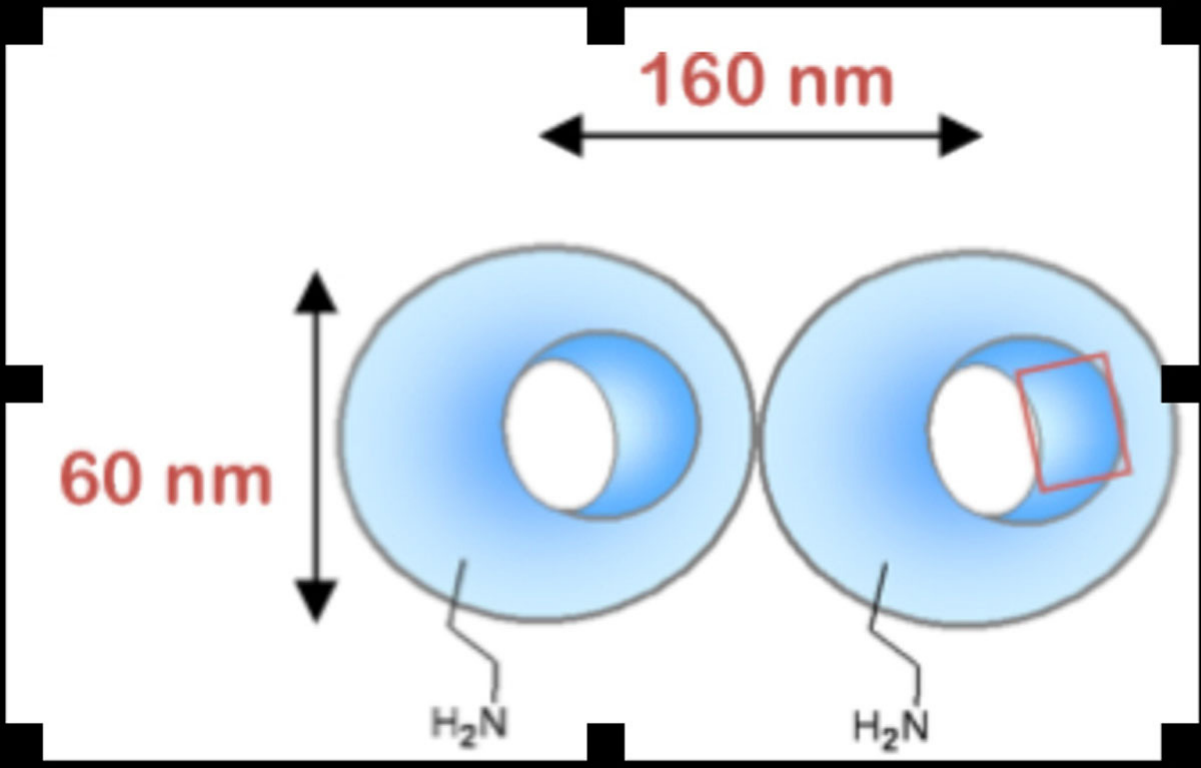
Cartoon depiction of NanoBialy based on hydrodynamic dynamic light scattering measurements. The particles appear biconcave or bialy-shaped, much like erythrocytes. The high surface-to-volume aspect of the particle is densely decorated with Mn-porphryn chelates.

**Figure 2:**
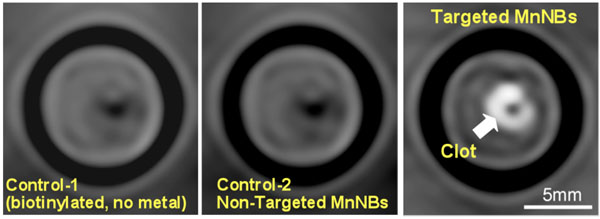
MRI images of fibrin-targeted nanobialys (right) or control nanoparticles bound to cylindrical plasma clots measured at 3.0T. The ionic and particulate r1 relaxivities of serially diluted nanobialys at 3.0T were 3.1 ± 1.1 (s ● mmol [Mn])-1 and 512,863 ± 8408 (s ● mmol [nanobialy])-1 respectively.

Mn-nano-bialys were 190nm ± 5nm with polydispersity of 0.26±0.01. (Fig 1) In the hydrated state, Manganese content was 25.6 ± 03 µg/mL by ICP OES, i.e., 165,000 Mn(III) per nanobialy. The particulate relaxivities of the MnNB were high, r1=612,307±7213 and r2= 866,989±10704 (s●mmol [nanobialy])-1 measured at 1.5T (25°C), with ionic r1 and r2 relaxivities of 3.7±1.1 and 5.2±1.1 (s●mmol [Mn])-1, respectively. MR imaging of MnNB targeted to fibrin clot phantoms showed clear contrast enhancement, while control clots had no (p<0.05) contrast change (Fig 2).

## Conclusions

Fibrin-specific MnNBs are a novel, high relaxivity, non-gadolinium, molecular imaging agent that offers a sensitive noninvasive MR imaging approach for diagnosis of ruptured atherosclerotic plaques.

## Funding

NIH.

